# The Importance of Psychometric and Physical Scales for the Evaluation of the Consequences of Scars—A Literature Review

**DOI:** 10.3390/clinpract13020034

**Published:** 2023-03-03

**Authors:** Gabriel Mihai Mekeres, Camelia Liana Buhaș, Andrei Nicolae Csep, Corina Beiușanu, Gineta Andreescu, Paula Marian, Cornel Dragoș Cheregi, Radu Fodor, Felicia Manole

**Affiliations:** 1 Doctoral School of Biomedical Sciences, University of Oradea, Faculty of Medicine and Pharmacy, 410087 Oradea, Romania; 2 Morphological Disciplines Department, Faculty of Medicine and Pharmacy, University of Oradea, 410087 Oradea, Romania; 3 Department of Legal Medicine, County Clinical Emergency Hospital of Oradea, 410169 Oradea, Romania; 4 Medical Disciplines Department, Faculty of Medicine and Pharmacy, University of Oradea, 410087 Oradea, Romania; 5 Surgical Disciplines Department, Faculty of Medicine and Pharmacy, University of Oradea, 410087 Oradea, Romania

**Keywords:** scars, psychometric scales, physical assessment scales, aesthetic prejudice

## Abstract

Background: Scars affect patients after trauma, burns, or surgical procedures and can generate both physical and psychosocial changes. The aesthetic damage represents the modification of a person’s physical appearance, in its bodily integrity, causing numerous sufferings and determining social or economic consequences. The aim of this research is to evaluate the advantages and disadvantages offered by the available psychosocial and physical scar scales in assessing the physical, aesthetic, psychosocial, and juridical consequences of scars. This will aid to inform medical examiners about the most valued existing scales to allow them to select the most appropriate instrument to manage their patient. Methods: A broad search of relevant scientific studies on the psychosocial determinants of post-traumatic and surgical scars was conducted by using the following international database tools: PsycINFO, MedLine Social Science Index, Scopus, Web of Science, published from 1960 until 2022. Results: We analyzed 63 scientific studies to assess the advantages and limits of several psychometric and physical scar evaluation scales. Conclusions: The researchers analyzed in this review highlight the advantages and limitation of existing instruments, evidencing the demand for future scar evaluation instruments and a scar-assessing algorithm that takes into account the physical, aesthetic, psychosocial, and legal consequences of scars.

## 1. Introduction

Traumas on the skin and mucous membranes, burns, or surgical procedures are healed by scarring, which may generate physical and psychosocial changes as well [1,2,3,4]. Several modalities have been developed to quantify scars in order to establish the response to treatment and to evaluate the physical component of the results, but the psychological component is little studied [5,6]. The most used scar assessment scales are the Vancouver Scar Scale (VSS) and Patient and Observer Scar Assessment Scale (POSAS) [7,8]. Scar measuring scales have difficulties in assessing the role of mental suffering induced by the process of their formation, which sometimes generates a change in the physiognomy or even alters the victim’s aesthetic perception of his/her own body. There are multiple consequences of scars such as pain, itching, aesthetic discomfort, and prejudice, or even functional impairment, mainly in the periarticular surface, that can determine physical, aesthetic, psychosocial, and juridic repercussions. Many studies refer only to the epidemiology and management of scars, while very few are interested in developing methods to objectify the consequences of scars [9]. Scar management should be approached by taking into account five main areas of impact on patients’ needs: physical comfort and function; acceptability for himself and others; social integration; trust in the healing process; emotional well-being. Medical staff should be available to develop proper management and help to reduce patient suffering [10]. One juridical repercussion is the aesthetic damage that represents the changes of a person’s physical appearance, in its bodily integrity, which causes numerous sufferings and which will have social or economic consequences [11,12,13]. Moreover, scars remain a trauma marker that represent an element of identification of a person, provides information about the nature of the weapon that cased it, the age of the scar, and can be used as evidence in court [14,15]. However, the individual can adopt coping behavior toward the scar-triggering event without affecting their body image and relationships with others through internalization of the scar [11]. If touching or observing the scar constantly reminds the person of the traumatic event and negatively influences their daily activities, it is considered that the patients have not internalized the scar and need a medico-legal examination [16]. Some psychosocial effects of scars are described by public or social stigma that occurs when a society has a belief about a certain group of people, resulting in a negative emotional impact and/or discrimination, feelings of hopelessness, fear, loss of identity, low self-esteem, guilt, isolation, anxiety, and depression [17,18]. Depression and post-traumatic stress disorder were evidenced in 13–45% of cases according to scientific studies in patients with burns [19]. Social problems include difficulties in sexual life and social interactions. The quality of life appears to be initially lower in scarred patients compared to the general population [20]. The main objective of plastic surgeons and dermatologists should be the improvement of the scar’s appearance and therefore to enhance the patient’s well-being. The medical examiner should refer the patient to plastic surgery to correct the physical aspects of the scar and to follow the patient’s evolution over time to decide if the patient has internalized the scar or if there is an aesthetic damage [21,22].

The aim of this research is to evaluate the advantages and disadvantages offered by the available psychosocial and physical scar scales in assessing their physical, aesthetic, psychosocial, and juridical consequences. This will aid to inform medical examiners about the most valued existing scales to allow them to select the most appropriate instrument to manage their patient.

## 2. Methods

A broad search of relevant scientific studies on the psychosocial determinants of post-traumatic and surgical scars was conducted by using international database tools: PsycINFO, MedLine Social Science Index, Scopus, Web of Science. We researched these databases for articles published from 1960 until 2022 discussing psychosocial and physical scales which are still in use. Keywords included in the process of surveying the specialized literature were as follows: psychosocial scar scale, physical scar scale, hopelessness depression, social support scale, aesthetic prejudice, scar consequences. An important step was to establish the selection criteria for the inclusion and exclusion of the studies identified in the international specialized literature. The articles were introduced in Microsoft Excel, then sorted by title and author’s name; duplicates were removed semiautomatically. Inclusion criteria were as follows: systematic reviews and literature reviews, and full text original articles, published in English in international journals as full text that measure the appraisal of surgical and traumatic scars, and developed and validated tools in the surgical and traumatic scar population. Exclusion criteria for the study were as follows: qualitative studies that reported data on scarring in children, studies that did not contain a comparison group, studies that were not published in English, conference abstracts, and case reports.

**Figure 1:**
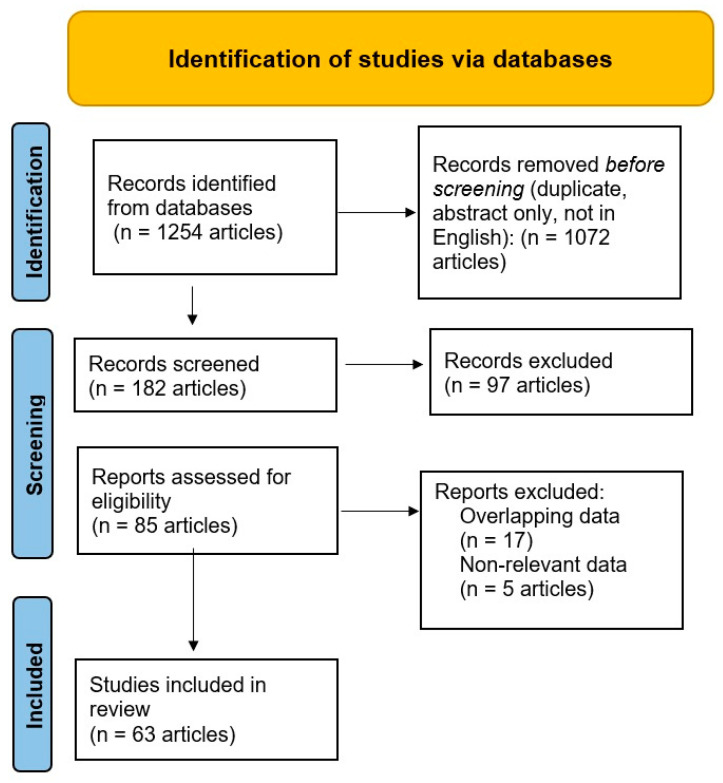
PRISMA flow diagram of the selection of medical articles [23].

The review identified 1254 articles in the employed databases; of them, 1072 were removed before screening, Figure 1. Throughout the screening process, the remaining 182 articles were manually reviewed by GMM, and further assessed by another two authors (ANC and FM). As a consequence, the other 97 records were excluded (no full-text available in English, modified version of scar rating scale). The remaining 85 articles were reanalyzed, and after excluding another 22, the remaining 63 articles were included in this review. The GMM performed the final manual selection of the studies we analyzed.

## 3. Results

Aiming to synthetize the information gathered from the 63 analyzed scientific articles, of which 8 were meta-analyses, 8 reviews, and 47 original articles, we divided them into three categories depending on the type of employed scales: physical, psychosocial, and combined methodology. The tables were structured based on the year of publication of the presented scales, starting with the most recent one.

### 3.1. Scales for Physical Assessment of Scars

The scales that evaluate the morphological characteristics of scars were designed to assess their appearance in response to the treatment, but these scales are observer-dependent, most of them only considering clinical aspects such as scar height or thickness, surface, texture, flexibility, vascularity, and pigmentation (Table 1). The scar rating scales are frequently used in scientific research and are beneficial for the study of small, linear scars, the main disadvantage being the minimal utility of evaluating large scars and their functional effects [24].

One of the first methods, the esthetic method, was proposed by Greff and Hodin, and quantifies the aesthetic damage according to the morphological characteristics of the scar. It is a laborious method, difficult to be utilized because it divides the face into 122 facial sectors, but a lesion can affect one or more sectors. To determine the degree of disfigurement, each sector is scored according to several coefficients, then a calculation is made for all affected sectors. The aesthetic coefficient (Cs) is calculated by multiplying the value of each coefficient for each affected sector, the total aesthetic coefficient (Ct) resulting from the sum of all aesthetic coefficients for the affected sectors. The maximum aesthetic deficit is 100% and corresponds to a total destruction of the face. The main counter-arguments for the use of the esthetic method are as follows: the division of the face into too many sectors (122), to which the associated parameters are added; the role of the forensic doctor in assessing the damage could be replaced by entering the data into an image processing software with the exact calculation of the damage according to the parameters introduced and analyzed; it does not quantify all body areas [50,51].

VSS, also called the Burn Scar Index, a method of measuring scars first described by Sullivan in 1990 [41], is probably the most recognized method of evaluating scars formed after burns. The VSS is a scale that assesses risk factors for the development of hypertrophic scars and the effectiveness of their treatment. This scale analyzes scar vascularity, pigmentation, foldability, and height to diagnose hypertrophic scars [43,44].

POSAS, proposed by Draaijers et al. [28] in 2004, allows a structured clinical evaluation of scar quality, reflecting both the observer’s and the patient’s opinions. This tool has been shown to be consistent and reliable for burn scars. The characteristics of scars such as vascularity, pigmentation, firmness, pliability, affected area, and scar height are scored. The POSAS is a standardized and validated instrument in the clinical, objective assessment of scars, also taking into account the patient’s symptoms related to the scar, such as pain and pruritus, which were not considered in previous scales [29,52].

### 3.2. Psychometric Scales for the Assessment of Scars

The psychosocial components and consequences of the presence of a scar are represented by the internalization of this particular scar, the social significance of the aesthetic injury, its experiences, the perceived social and family support at the time of the traumatic event, and the stage of personality development [16]. By inserting these psychosocial considerations that guide scar treatment decisions, future scar assessment tools can accomplish far more than characterizing the clinical aspects of scars (Table 2) [47].

MPIS was validated in 2021 following a study including 204 participants presenting scars. The lot, aged between 18 to 81 years old, was split into two groups, of which 105 were women and 99 men. Each individual had to answer 15 questions marked from 1 (not agree) to 5 (totally agree) using the Likert scale. Therefore, the MPIS score was a total sum anywhere between 15 to 75. Moreover, proved to have a good consistency (Cronbach, 0.943), the scale highlights the individual’s scar awareness, gender, the morphological aspect of the scar, as well as its social and professional impact on the affected patients [16].

Developed by Zimet, Dahlem, Zimet, and Farley, (1988), MSPSS comprises 12 items based on 3 main factors, such as family, friends, and significant people, each item being designed according to the above factors. Showing a high internal consistency (alfa Cronbach 0.91) [58], the scale was built to reflect the highest social support known based on the highest scoring.

HDSQ (Metalsky and Joiner,1997) was introduced to help monitor hopelessness depression [62]. The tool is based on 32 items, allowing the examiner to observe various symptoms on individuals. HDSQ’s structure is similar to DBI’s, where each symptom is measured in a group of four items. Therefore, the tool totals a number of eight subscales (Motivational Deficit—retarded initiation of voluntary responses, Interpersonal dependency, Psychomotor retardation, lack of energy, Apathy/anhedonia, Insomnia, Concentration difficulty and Suicidality), each element including four items, each measuring a different symptom of hopelessness depression. The above scale showed a high internal consistency (alfa Cronbach 0.70 and 0.93) [62,63].

### 3.3. Scales for Physical and Psychosocial Assessment of Scars

The Scar-Q Aesthetics is a new tool that evaluates all types of scars, consisting of three subscales: the appearance scale (19 items) that assesses size, width, color, and contour of scar; the scar symptoms scale (17 items); and the psychosocial impact scale (12 items). Each part is a set of items with 4-point responses (1 = most favorable–4 = least favorable). The sum of the items measures each subscale. If the patients have more than one scar, instructions elucidate that the answer should be thinking about the scar that bothers them the most (Table 3) [66].

PSAQ is a valid measure developed by Durani et al. in 2009. This reliable measure took into account physical, aesthetic, and psychosocial consequences of surgical scars. Except the Symptoms subscale, the other four subscales indicated a high internal consistency (Cronbach α, 0.73 to 0.93) [72].

## 4. Discussion

It is highly important that the scales for scar evaluation should be well known by clinicians considering the multiple morpho-functional, aesthetic, psycho-social, and legal consequences of scars. Acknowledging the benefits and downsides of these scales could help clinicians choose the most appropriate method of approach.

The aesthetic method derived from Greff’s and Hodin’s methods [50], VSS [41], MSS [33], and SBSES [24] analyzes the morpho-functional and topographic aspect of the scars; POSAS additionally assesses pain and itching symptoms. The POSAS is a standardized and validated instrument in the clinical, objective assessment of burn scars, also taking into account patient symptoms related to the scar, such as pain and pruritus, which were not considered in previous scales [28]. The VSS is a scale that assesses risk factors for the development of hypertrophic scars and the effectiveness of treatment on burn scars [41,69]. The disadvantage of these clinical tools is the lack of a psychosocial and juridical component in assessing scars. The Hamilton Scale [37] and SSS [39] assess scars through photographs.

The evaluation of the aesthetic prejudice remains a component of the medical examiner’s activity where the expected criteria are insufficiently outlined as a consequence of the weight of the subjective elements related to the traumatized victim. The difficulties reside in the fact that moral suffering cannot be objectively evaluated, which causes impediments in the stability of the compensation value to those qualified [51]. The aesthetic method derived from Greff’s and Hodin’s method, used to establish the aesthetic damage, presents multiple limitations (the division of the face in 122 sectors plus associated parameters, lack of interpretation of the result) [11,50]. MPIS includes the psycho-social component in scar evaluation in its legal aspect, making the obtained scale score influence the existence of aesthetic damage, disfigurement, and scar internalization [16]. To internalize the scar more easily, people try to hide the scar; this makes them unsociable and interferes with their communication skills, personal relationships, professional life, and agreed activities [11,16].

Scars can negatively affect body image and self-confidence. Scars have been stigmatized in society since cartoons and horror movies depict them as embodying a bad person. Moreover, scars can also cause functional disorders and have professional and financial repercussions [76,77]. From a physical point of view, people with scars can develop psychiatric conditions manifested by depressive symptoms, anxiety, shame, and sometimes anger and aggression or avoidance behavior. After the occurrence of the traumatic injury, the social support of the family and those close to them is important in order to internalize the scar as easily as possible in order to receive psychological acceptance [78]. Facial scars cause a high level of anxiety and decreased self-confidence [20]. Brown et al. (2010) examined the physical comfort and functionality of patients with scars as well as their confidence, the way they manage their affections, the way they accept themselves and others, their wellbeing, and social interactions. The results showed that more than half of the participants were unhappy with their scar appearance, about 20% of them developed anxiety, and 50% felt their private and intimate relationships were compromised [35]. The above-mentioned psycho-social consequences relate to other studies in which scarred patients felt condemned and labelled, leading them to social isolation, difficulties to adapt in society, and the need to hide their scars [22]. MBSRQ and HDSQ are successfully used in body image research and scar assessment.

Social problems include difficulties in sex life and social interactions. Quality of life appears to be lower in patients with burns compared to the general population. Mental health problems are more significant than physical problems. For many years, quality of life was reported to be quite good [79,80,81]. MSPSS can be used to assess the psychosocial effects of scars, mediating variables such as low social support, avoidant coping styles, and personality traits negatively that affect postburn adjustment [58]. Optimizing adaptation after the formation of post-traumatic scars requires the support of family and friends who promote socialization, acceptance of the scar, and, if necessary, special psychological or psychiatric help. Most studies were conducted using physical assessment scales. However, PSAQ and Scar Q explored the psychosocial components of scars and the notion of recognition and acceptance [66,72].

Kim et al. (2022) published a study in which 60 patients with keloid scars were investigated. The patients completed all three subscales of the SCAR-Q (symptoms, appearance, and psychosocial impact). The study’s aim was to compare pre- and postoperative scar perception. The study showed that the patients felt a significant improvement in all parts of the SCAR-Q [71].

Moreover, a study comparing laparoscopic cholecystectomy to single incision intervention, conducted by Ostlie et al. (2013) across 60 patients, aimed to establish the possible existing cosmetic advantages, analyzing the difference between the two procedures. Each patient was asked to complete a PSAQ survey containing four parts: Appearance, Satisfaction with Appearance, Consciousness, and Satisfaction with Symptoms. The last subscale was omitted from the analysis because of deficient reliability [72]. The results of this research showed that both the appearance and the number of scars is important, the aesthetic benefit favoring the one-site approach. Another study used the PSAQ to evaluate improvements of conscious scar location after breast cancer surgery, comparing the preferred port between arm and chest. In conclusion, although most of the patients were conscious of their scar location, the arm site was preferred rather than the chest [73].

Mundy et al., in a systematic review published in 2016, analyzed four patient-reported outcome (PRO) instruments POSAS, Bock quality of life questionnaire for patients with keloid and hypertrophic scarring (Bock), PSAQ, and patient-reported impact of scars measure (PRISM). All used tools, measured symptoms, and psychosocial aspects of scars; additionally, PSAQ had physical assessment. The limit of this tool was the lack of legal aspect [74].

In this day and age, we are lacking a scale assessment tool that would appreciate all physical, aesthetic, psycho-social, and legal aspects of scars. Following this research, we appreciate that a scar evaluation algorithm containing several scales applied according to the age of the scar (0–6 months and >6 months) would be useful. Firstly, following a plastic surgery clinician review, the injury would be evaluated from a morpho-functional point of view and initial treatment would be commenced. Therefore, SBSES can be used at this stage, having the advantage of cosmetically assessing the outcomes of a wound up to 5–10 days from the injury until the sutures are removed. Moreover, if the injury is located on victim’s face, the Scar-Q scale is recommended, having higher outcomes on facial aesthetic treatments. Secondly, a medical examination will be required to evaluate the gravity of the injury from its legal aspect, to determine the psychological impact, and, therefore, help identify post-traumatic stress disorder (PTSD). HDSQ and MSPSS can be used at this stage to help identify the most appropriate psychological support and ensure an adequate psychological or psychiatric evaluation. Nonetheless, if the scar is more than 6 months old, the plastic clinician could use the POSAS and Scar-Q or PSAQ scales that take into account both patient and observer points of view, plus psychosocial aspects. A further medical legal consultation must be considered to determine the legal repercussions of the scar; however, using MPIS can still aid the internalization of the scar without legal consequences, aesthetic prejudice, or disfigurement with legal repercussions.

## 5. Conclusions

In the literature, little is known about how scars affect patients’ lives. This review debates the advantages and limitation of some existing instruments, focusing on demand for future a scar instrument development or a scar evaluation algorithm that takes into account the physical, aesthetic, psychosocial, and legal consequences of scars.

## Figures and Tables

**Table 1:** Scales for physical assessment of scars.

No.	Scale	Year	Scar Consequence	Advantage	Disadvantages	References
1.	Stony Brook Scar Evaluation Scale (SBSES)	2007	-aesthetic; (6 items score: 0—worst to 5—best).	-research measuring short-term (5–10 days) wound outcomes; -cosmetic outcome of wounds (5–10) days after injury until suture removal; -easy to use.	-limited applicability; -psychosocial and juridical consequences are missing.	[24,25,26,27]
2.	Patient and Observer Scar Assessment Scale (POSAS)	2004	-physical; -aesthetic (2 subscale score between 1–10).	-assess vascularity, thickness, pigmentation, foldability, relief and pain, itching; -first scale that takes into account observer and patient ratings; -measures scar quality; -evaluates all types of scars.	-lack of psychological and juridical determined by the scar.	[28,29,30,31,32]
3.	Manchester Scar Scale (MSS)	1998	-physical (4 parameters, score 4 best outcome-14 severe scar); -aesthetic.	-assess color, shine, contour, distortion); -wider scar applicability; -suitable for surgical scars.	-not used in research; -psychosocial and juridical effect are missing.	[33,34,35,36]
4.	Hamilton Scale	1998	-physical; -aesthetic (photographs).	-using only photographs; -thickness, surface irregularity, vascularity, and scar size; -increased ability even in inexperienced persons.	-not based on actual scars; -psychological and juridical damage missing.	[37,38]
5.	Seattle Scar Scale (SSS)	1997	-physical; -aesthetic (24 standard color photographs).	-color photographs; -assess: surface, height, thickness, and color differences between the scar and adjacent normal skin, using a numerical scale; -distinguish different types of scars; -scar severity.	-did not include psychological and juridical damage.	[39,40,41]
6.	Vancouver Scar Scale (VSS)	1990	-physical (the first scar evaluation scale applied: score 0–13); -aesthetic (score: 0 and 13 points).	-thickness, height, vascularization, pliability, pigmentation; -the most frequently used scar scale; -evaluates post-burn scars; -widely used in clinical practice and research; -document change in scar appearance.	-lacks patient perception; -semi-quantitative approach to subjective characteristics; -inaccurate reproducibility evidence in the case of large or irregular scars; -does not take into account the physical and psychosocial sequelae of scars.	[42,43,44,45,46,47,48,49]
7.	The aesthetic method derived from Greff’s and Hodin’s methods	1973	-aesthetic; -physical (divides the face into 122 sectors plus correction coefficients: coefficient C, coefficient F for correcting a fracture, coefficient R for relieves, coefficient P—the plastic coefficient, coefficient c—the coefficient of color, T—texture coefficient).	-assess the face: anterior, lateral view, and correction coefficients.	-the division of the face, the front plane and its side planes into too many sectors plus associated parameters; -does not quantify all body areas; -multiple calculations; -final formula has no interpretability for aesthetic damage; -psychosocial sequelae are missing.	[11,50,51]

**Table 2:** Psychometric scales for the assessment of scars.

Nr.	Scale	Year	Scar Consequence	Advantages	Disadvantages	References
1	Mekeres’ Psychosocial Internalization Scale (MPIS)	2017	-psychosocial; -juridical; (15 items, score between 15 and 75 points).	-assesses internalization, aesthetic prejudice, and disfigurement; -presence or absence of aesthetic prejudice; -easy to use.	-used in post-traumatic injuries; -lack of physical assessment.	[11,16,22]
2	Multidimensional Body-Self Relations Questionnaire (MBSRQ)	1990	-psychosocial; (69 items, subscales: appearance evaluation, appearance orientation, overweight preoccupation, body areas satisfaction scale.	-self-report inventory; -body image construction; -widely, successfully used in body image research.	-no physical or juridical assessment; -long examination time.	[53,54,55,56,57]
3	Multidimensional Scale of Perceived Social Support (MSPSS)	1988	-psychosocial; (12 items, 3 subscales: family, friends, and significant others).	-a self-report scale; -determine social support, appreciation, acceptance, and self-awareness, emotional support in times of crisis; -follows the social perception of friends, family, and significant others.	-missing physical and juridical elements in the examination of the scar.	[18,22,58,59,60,61]
4	Hopelessness Depression Symptom Questionnaire (HDSQ)	1987	-psychosocial; (32 item, 8 subscales).	-supports the existence of hopeless depression, a subtype of depression; -assessment of hopeless depression and hopelessness theory of depression; -clinical tool that takes into account the fact that each minor subscale measures a specific symptom; -allows clinicians and patients to test depression symptoms separately and not just a global score (to reflect the severity of the disorder).	-missing physical and juridical elements in the examination of the scar; -long examination time.	[20,22,62,63,64,65]

**Table 3:** Scales for Physical and Psychosocial Assessment of Scars.

No.	Scale	Year	Scar Consequence	Advantage	Disadvantages	References
1.	SCAR-Q Aesthetics	2018	-aesthetic; -physical; -psychosocial (48 item, 3 subscale).	-evaluate all types of scars; -3 domains: appearance, health-related quality of life, and adverse effects.	-the juridical aspects missing.	[66,67,68,69,70,71]
2.	The Patient Scar Assessment Questionnaire (PSAQ)	2009	-aesthetic; -physical; -psychosocial.	-evaluate surgical scars; -5 subscales: appearance, symptoms, satisfaction with appearance, and with symptoms, consciousness.	-do not evaluate traumatic scars; -reliable for linear scars; -the juridical aspects missing.	[72,73,74,75,76]

## Data Availability

Not applicable.
